# The framing effect and skin conductance responses

**DOI:** 10.3389/fnbeh.2015.00188

**Published:** 2015-08-05

**Authors:** Patrick Ring

**Affiliations:** ^1^Kiel Institute for the World EconomyKiel, Germany; ^2^Institute of Psychology, Kiel UniversityKiel, Germany

**Keywords:** electric shock, emotion, framing effect, judgmental heuristics, skin conductance responses

## Abstract

Individuals often rely on simple heuristics when they face complex choice situations under uncertainty. Traditionally, it has been proposed that cognitive processes are the main driver to evaluate different choice options and to finally reach a decision. Growing evidence, however, highlights a strong interrelation between judgment and decision-making (JDM) on the one hand, and emotional processes on the other hand. This also seems to apply to judgmental heuristics, i.e., decision processes that are typically considered to be fast and intuitive. In this study, participants are exposed to different probabilities of receiving an unpleasant electric shock. Information about electric shock probabilities is either positively or negatively framed. Integrated skin conductance responses (ISCRs) while waiting for electric shock realization are used as an indicator for participants' emotional arousal. This measure is compared to objective probabilities. I find evidence for a relation between emotional body reactions measured by ISCRs and the framing effect. Under negative frames, participants show significantly higher ISCRs while waiting for an electric shock to be delivered than under positive frames. This result might contribute to a better understanding of the psychological processes underlying JDM. Further studies are necessary to reveal the causality underlying this finding, i.e., whether emotional processes influence JDM or vice versa.

## 1. Introduction

Evaluating the probability of uncertain events is fundamental for human judgment and decision-making (JDM). In order to assess the likelihood of uncertain outcomes, people often apply rules of thumb, so-called judgmental heuristics. On the one hand, heuristics might be advantageous by shortening the decision process or by shifting attention to important aspects of a choice problem. On the other hand, they can lead to discrepancies between an individual's assessment of a situation and objective measures (Tversky and Kahneman, 1974). For example, when people estimate the likelihood of an event, they are typically influenced by current news reports. Media coverage, however, is usually high for rare events, but low for common events, and therefore not representative of the probability of an event. Consequently, people tend to overestimate the probability of rare events, such as airplane accidents, and underestimate the probability of common events, such as car accidents. This mental short-cut is known as the availability heuristic (Tversky and Kahneman, 1973).

Studying judgmental heuristics is an important field of research for at least two distinct reasons. First, they suggest insights into the psychology underlying human judgment and thereby improve our understanding of individual decision-making (Tversky and Kahneman, 1983). These insights might provide more realistic foundations for models of human behavior (e.g., Kahneman and Tversky, 1979). Second, they can explain aggregate market results, e.g., overconfidence potentially explains excessive trading in stock markets (Barber and Odean, 1999). While traditionally it has been proposed that cognitive processes are the main driver to reach a decision, growing evidence stresses the importance of emotion on JDM (Ochsner and Phelps, 2007; Angie et al., 2011; Phelps et al., 2014; Lerner et al., 2015).

Damasio (1994), for example, argues that emotional body signals play an important role in decision-making. According to his theory, changes in the somatic state of an individual do not only accompany human behavior, but they have direct influence. This is supported by empirical evidence from studies using the Iowa Gambling Task (IGT). In the IGT, patients with damage to the ventromedial sector of prefrontal cortices and healthy control participants choose one card from four available decks. While two decks are advantageous because they have a positive expected value, the other two decks are disadvantageous in the sense that they have a negative expected value. In the course of the experiment, healthy participants learn to choose cards from the advantageous decks. At the same time, they produce anticipatory skin conductance responses (SCRs) preceding disadvantageous decisions even before they are able to verbally express the rule underlying this task. In contrast, patients do not follow an optimal strategy and also lack the aforementioned body reactions. Some of them, however, are able to verbally identify the advantageous decks at the end of the experiment. Based on this finding, Damasio and colleagues conclude that overt reasoning might not be enough to make advantageous decisions and that emotional reactions appear necessary (Bechara et al., 1994, 1996; Damasio et al., 1996; Bechara et al., 1997).

Furthermore, Slovic and colleagues (Finucane et al., 2000; Slovic et al., 2004, 2005) claim that affect, i.e., the experience of emotion, has an impact on JDM. They argue that positive feelings toward a stimulus (i.e., positive affect) would lead to a lower risk perception, even if this does not match objective probabilities. Negative feelings (i.e., negative affect), by contrast, would lead to the opposite effect. This claim is supported by several studies (Fischhoff et al., 1978; Slovic et al., 1991; McDaniels et al., 1997) that find that activities with high perceived benefits are considered to be less risky, e.g., vaccines or antibiotics. On the other hand, activities with low perceived benefits are considered to be more risky, e.g., smoking or alcohol consumption.

Empirical studies highlight the interplay between emotional arousal and JDM in various settings. For example, Van't Wout et al. (2006) find that respondents' SCRs in the Ultimatum Game are higher for unfair than for fair offers. Higher emotional activity is also associated with higher rejection rates for unfair offers. Schmidt et al. (2013) show that participants with low resting arousal are more likely to engage in risky gambling behavior than participants with high resting arousal. This finding is in line with theories stating that lowly aroused individuals might try to reach an optimal level of arousal through participation in risky activities. In studies by Ariely and Loewenstein (2006) and Ditto et al. (2006), sexual arousal influences the individual likelihood to pursue risky activities. Furthermore, emotional arousal has an impact on inter-temporal decision-making (Sohn et al., 2015) and also on aggregate market results (Hirshleifer and Shumway, 2003).

Recent studies address the interplay between emotion and judgmental heuristics from different perspectives. Firstly, brain imaging studies indicate that brain areas, which are typically associated with emotional processes, are relevant for the individual susceptibility to judgmental heuristics. Clark et al. (2014), for example, analyze brain regions related to the “gambler's fallacy.” The authors find that participants with injuries to the insula region, an area of the brain linked to emotion (Singer et al., 2009), are less prone to this judgmental heuristic. Deppe et al. (2005) find a correlation between the degree of an individual's susceptibility to the framing effect and activity in the medial prefontal cortex. This area of the brain is supposed to be important in processing emotional information (Bechara et al., 2000). Similarly, De Martino et al. (2006) identify a correlation between increased activation in the amygdala and different risk-taking behavior under gain and loss frames. Among other things, the amygdala is relevant for the identification of emotionally relevant stimuli (Davis and Whalen, 2001).

Secondly, the effect of induced emotion on the individual susceptibility to judgmental heuristics has been assessed. Ma et al. (2015) show that negative emotion alters risk preferences in a decision-making task under positive frames but not under negative frames. Under happy induced mood, participants exhibit greater framing effects than under sad induced mood (Stanton et al., 2014). Moreover, Cassotti et al. (2012) find that under a positive emotional context, the framing effect disappears. Different dimensional representations of emotion might explain these findings^1^. Additionally, in a study by Cheung and Mikels (2011), emotional regulation leads to less risk-taking behavior under both gain and loss frames. Reliance on emotion, however, leads to a similar level of framing effects compared to a control condition.

Thirdly, Sarlo et al. (2013) show an effect of framing on emotional body reactions. In particular, the authors study how framing of offers in the Ultimatum Game affects autonomic responses (heart rate and SCRs). Autonomic response patterns in men are different when offers are presented under gain (“I give you”) and loss framing (“I take”). Women's autonomic responses are not affected by different frames.

Based on the stated literature above, which indicates a relation between emotion and judgmental heuristics, this paper addresses the impact of framing on emotional body reactions in a risky situation. In general, framing describes a judgmental heuristic where individuals react systematically different to the same choice problem depending on how it is presented. For example, Tversky and Kahneman (1981) explore how framing affects participants' decisions in a hypothetical life and death situation. In their choice problem, participants choose between two treatments for 600 potential victims of a deadly disease. It is predicted that treatment A would lead to 400 deaths, whereas treatment B has a 33% chance that no one would die, but a 66% chance that everyone would die. This hypothetical choice is presented either in a positive frame emphasizing how many people would survive or in a negative frame emphasizing how many people would die. In a positive frame, 72% of the participants choose treatment A. In a negative frame, by contrast, only 22% of the participants opt for treatment A. In this example, participants are risk-seeking in the loss-domain but risk-averse in the gain-domain. This finding violates rational economic theory, which assumes that changes in frames do not alter behavior in a systematic way. Framing effects are robust findings that are reported in different settings (e.g., Johnson et al., 1993; Gächter et al., 2009).

In the following experiment, I adopt the logic of *attribute framing* to a risky situation^2^. Participants are exposed to different probabilities of receiving an unpleasant electric shock that are either framed with a negatively valenced proportion (“You receive an electric shock with ..% probability.”) or with a positively valenced proportion (“You do not receive an electric shock with ..% probability.”). The anticipatory reactions measured by integrated SCRs (ISCRs) while waiting for an electric shock to be delivered are used as an indicator for participants' emotional arousal (Boucsein, 1992). This indicator serves as a measure for the subjective evaluation of a situation, and it is compared with objective measures. Due to framing effects, negatively valenced frames, which emphasize electric shock occurrence, might lead to higher emotional arousal than the corresponding positively valenced frames, although electric shock probability is the same. I tested the following hypothesis regarding participants' emotional arousal measured by ISCRs:

Hypothesis: Participants show higher anticipatory ISCRs when electric shock probabilities are presented in negative frames than when they are presented in positive frames.

## 2. Materials and methods

### 2.1. Subjects

Forty undergraduate psychology students (gender: 25 female, 15 male; age: *M* = 22.1 years, *SD* = 3.1) took part in the study in exchange for course credit points. Participants gave written informed consent and could decide to discontinue participation at any time. The research design was approved by the Ethics Committee of the German Psychological Society and performed in agreement with the Declaration of Helsinki.

### 2.2. Experimental design

Each participant was exposed to 24 situations of potentially receiving an electric shock. Electric shock probabilities were presented on a computer screen as either “You receive an electric shock with ..% probability.” or “You do not receive an electric shock with ..% probability.” While the first statement represents the negative frame, i.e., the probability of receiving an electric shock is emphasized, the latter statement represents the positive frame, i.e., the probability of not receiving an electric shock is emphasized. After presenting the electric shock probability, there was a 7-second (s) pause until the electric shock was applied or not with the previously stated probability. The outcome of this lottery was visualized by a red screen in the case of an electric shock and a green screen in the case of no electric shock. The next round started after 10 s. Three different electric shock probabilities were applied–20, 50, and, 80%–while each probability was presented four times in a positive frame and four times in a negative frame. The order of electric shock probabilities and their outcomes were randomized.

Participants were first instructed in written form and then verbally. In addition, participants completed a short questionnaire testing whether they could infer the probability of receiving an electric shock from both negative and positive framed statements. The average duration of one session was approximately 25 minutes (min). The software package Psychtoolbox-3 (www.psychtoolbox.org) running on MATLAB 7.1 (MathWorks Inc., United States) was used for stimulus presentation and response acquisition.

### 2.3. Electric shock stimulation

Electric shock was delivered via electric stimulation with a Canicom 800 (Num'Axes, France) (Levels of stimulation: 15; frequency of each single stimulation burst: 1.023 Hertz (Hz); duration of the whole stimulation burst: 115 ms; power range: 0.5–206 milliwatt). The stimulation was administered via two flat AG-AGCL electrodes of 10 millimeters (mm) in diameter being placed at the medial phalanges of digits II and III of the dominant hand. Individual levels of electric shock stimulation were calibrated using the following standard procedure: electric shocks were presented in an ascending series of intensity until the participant indicated that the electric shock was painful. Once a painful level was reached, the previous non-painful stimulation level was used during the experiment.

### 2.4. Psychophysiological measurements

I used a 16-channel bioamplifier (Nexus-16, Mind Media B.V., the Netherlands) and the corresponding recording software Biotrace (Mind Media B.V.) to record electrodermal responses. Two flat AG-AGCL 10-mm-diameter electrodes were placed at the medial phalanges of digits II and III of the non-dominant hand. In accordance with common recommendations (Roth et al., 2012), the electrode sites were prepared with an isotonic paste (TD-246, Discount Disposables, U.S.A.), and there was a 5-min pause between attaching the electrodes and starting recording. Skin conductance (SC) data were sampled at 32 Hz.

### 2.5. Data analysis

SC data were analyzed using Ledalab (www.ledalab.de) applying continuous decomposition analysis to disentangle phasic components from tonic activity (Benedek and Kaernbach, 2010). The ISCR, which is defined as the time integral of the phasic driver for a relevant time interval, was used as a measure for the phasic electrodermal response to a given stimulus. In order to account for the typical skewed distribution of the magnitude of electrodermal responses, individual ISCRs were standardized by the formula *Log ISCR* = *log*(1 + |*ISCR*|) × *sign*(*ISCR*) (Venables and Christie, 1980; Benedek and Kaernbach, 2011).

In order to measure sensitivity toward the likelihood of receiving an electric shock, ISCRs were computed for the 4-s window starting 3 s after revealing electric shock probability. I skipped the first 3 s to give participants enough time to realize the given electric shock probability and to discard any effect due to visual processing.

For ANOVA analysis, degrees of freedom were corrected by means of the Greenhouse-Geisser (G-G) method where necessary.

## 3. Results

**Figure 1:**
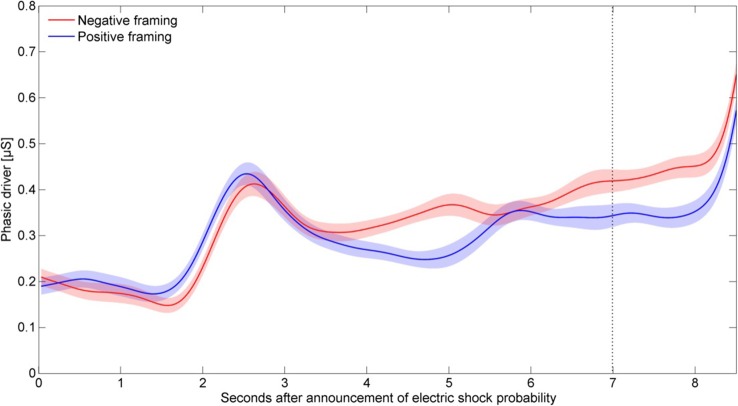
**Averaged phasic driver for positive vs. negative framing**. Shaded areas indicate the within-participant standard errors of the mean.

Figure 1 shows the averaged phasic driver for positive vs. negative framing over all electric shock probabilities. At 2.5 s after announcement of electric shock probability, SCRs reached a peak. The reaction in SCRs reflects visual processing (Boucsein, 1992). At approximately 3 s, the averaged phasic driver split for both conditions and the averaged driver for the negative framed electric shock probabilities remained above the averaged driver for the positive framed electric shock probabilities. Around 8.2 s, the electric shock was processed in the SC data.

**Figure 2:**
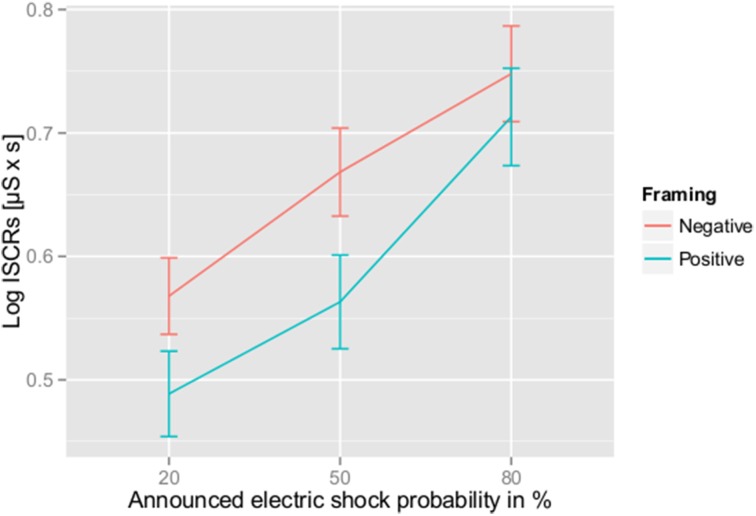
**Averaged ISCRs for positive vs. negative framing**. Error bars indicate the within-participant standard errors of the mean.

Additionally, Figure 2 shows Log ISCRs [microsiemens (μS) × s] for the 4-s time interval starting 3 s after the announcement of electric shock probability for all combinations of electric shock probabilities and framing. A Two-Way ANOVA for repeated measurements revealed a significant main effect for framing: $F_{\left(1,\;39\right)}=7.61,\text{p}=0.01,\eta_{\text{p}}^{2}=0.16$. The event type, i.e., the probability of receiving an electric shock, also had a significant main effect on Log ISCRs [G-G corrected $(\varepsilon =0.99)]:F_{\left(1.99,\;77.51\right)}=12.30,\text{p}<0.001,\eta_{\text{p}}^{2}=0.24$. This finding replicates a result from Ring and Kaernbach (2015). Please refer to the article for more detailed information. There was no significant interaction between framing and event type [G-G corrected $(\varepsilon \;=\;0.82)]:F_{\left(1.65,\;64.22\right)}=0.57,\text{p}=0.54,\eta_{\text{p}}^{2}=0.01$.

## 4. Discussion

As a result, I find evidence for emotional body reactions corresponding to the framing effect. Under negative frames, participants show significantly higher ISCRs while waiting for an electric shock to be delivered than under positive frames. This finding challenges the assumptions of rational economic theory, which state that changes in frames do not alter individual assessments of a situation in a systematic way. Furthermore, it is relevant, because it points toward a connection between emotion and JDM, which is also indicated in recent theories, e.g., the somatic marker theory by Damasio and colleagues (Bechara et al., 1994, 1996; Damasio et al., 1996; Bechara et al., 1997) or the affect heuristic by Slovic and colleagues (Finucane et al., 2000; Slovic et al., 2004, 2005). The following discussion has three main parts. The first part relates my result to previous research on judgmental heuristics and emotion. The second part concerns the experimental design and its limitations. Finally, the relation among judgmental heuristics, intuition, and emotion is considered.

While this study uses a psychophysiological measure to study judgmental heuristics, several studies applied brain imaging techniques (Deppe et al., 2005; De Martino et al., 2006; Clark et al., 2014). The general finding of these studies is that brain areas, which are typically associated with emotional processes, are relevant for the individual susceptibility to judgmental heuristics. The finding of this paper is in line with these studies, highlighting an interplay between emotion and judgmental heuristics. Recent studies by Cheung and Mikels (2011), Cassotti et al. (2012), Stanton et al. (2014), and Ma et al. (2015) have addressed the interplay between emotion and judgmental heuristics on the behavioral level. These studies analyze how induced emotion affects the individual susceptibility to judgmental heuristics and thereby behavior. Although these papers also indicate a link between emotion and judgmental heuristics, the perspective in this paper is a different one. In this paper, distinctive bodily states are elicited from a passive exposure to an aversive event. Information about the situations is positively or negatively framed, i.e., the effect of different formulations of the same content on body reactions is studied. This approach is most related to the previously mentioned study by Sarlo et al. (2013) showing different autonomic response patterns for men under gain and loss frames in the Ultimatum Game. Two main differences, however, exist. First, Sarlo et al. (2013) use monetary incentives to stimulate gains and losses. In this study, negative events are realized by means of receiving an unpleasant electric shock. Positive events are realized by means of not receiving an unpleasant electric shock. The choice of electric shocks instead of monetary stimuli is due to potential problems of simulating monetary losses in experimental setups. For a further discussion on this topic, see Berns et al. (2008). Second, outcomes in the study by Sarlo et al. (2013) are deterministic. In my study, the results are uncertain, i.e., whether participants in a given situation receive an electric shock or not. Real-world decisions are typically uncertain and rarely deterministic. Finally, this paper also relates to studies that show that emotional arousal impacts decision-making (Hirshleifer and Shumway, 2003; Ariely and Loewenstein, 2006; Ditto et al., 2006; Van't Wout et al., 2006; Schmidt et al., 2013; Sohn et al., 2015). The passive nature of my experimental design is discussed in the following part of the paper.

I measure body reactions while individuals assess the riskiness of a situation without active decision requirement. This was done, because as the experimenter I can not clearly detect the precise timing of a decision. Thus, differentiation between a pre- and post-decision phase would require some form of participants' behavior allowing participants to communicate that they have reached a decision. Preparation for motor activation and motor activation itself, however, could potentially contaminate recorded body reactions (Boucsein, 1992). Focusing on a passive situation in my design seems to be a more consistent approach. The described approach, however, has some limitations. First, decision phase and emotional body reactions are disentangled. Therefore, this design does not allow any statement regarding the causality of my finding, i.e., whether emotional processes influence JDM or vice versa. Second, my design does not identify to which extent a particular individual is biased by the framing heuristic. Therefore, I can only make aggregate conclusions. In essence, it is known from the related literature that people's decisions are influenced by framing effects, and I find on an aggregate level body reactions corresponding to this heuristic. It would be interesting, however, to see whether individuals, who are more heavily influenced by the framing heuristic, also show bigger differences in their emotional reactions. This question might be answered by testing individuals by the degree to which they are guided by this heuristic and then analyzing their emotional body reactions in the described way. Most studies on framing effects, however, apply between-participant designs (Tversky and Kahneman, 1981). An exception is a study by Stanovich and West (1998).

Finally, the relation among judgmental heuristics, intuition, and emotion deserves further attention. Heuristics are generally considered to be a form of intuitive decision-making, in the sense that these processes are beyond people's conscious awareness (Hogarth, 2010). Moreover, some authors consider affect, i.e., the experience of emotion, as typically the first and automatically evoked reaction toward a stimulus (“We do not just see ‘a house’: We see a *handsome* house, an *ugly house*, or a *pretentious* house” (Zajonc, 1980, p. 154). These first reactions are supposed to influence information processing and thereby JDM. If heuristics are guided by intuitive processes and emotions are typically the first reaction to a stimulus, it appears reasonable to assume that there should be a connection between judgmental heuristics and emotion. In this paper, I am able to identify emotional body reactions corresponding to the framing effect. These body reactions occur *before*, i.e., in anticipation of, a negative stimulus. This finding potentially motivates further studies analyzing causal links among judgmental heuristics, intuition, and emotion.

## Funding

The study is part of the project “Neurobiological Foundations of Economic Decision Making under Uncertainty and Excessive Risk Taking,” which is supported by the Leibniz Association (SAW-2013-IfW-2). The funders had no role in study design, data collection and analysis, decision to publish, or preparation of the manuscript.

### Conflict of interest statement

The author declares that the research was conducted in the absence of any commercial or financial relationships that could be construed as a potential conflict of interest.

## Footnotes

^1^Please refer to Kaernbach (2011) and Condon et al. (2014) on dimensions of emotion.

^2^According to Levin et al. (1998), framing effects can be divided into three distinct classes: *attribute framing, risky choice framing*, and *goal framing*. Please refer to the article for more detailed information.
